# Structure and function of molecular machines involved in deadenylation-dependent 5′-3′ mRNA degradation

**DOI:** 10.3389/fgene.2023.1233842

**Published:** 2023-10-09

**Authors:** Qi Zhao, Lorenzo Pavanello, Mark Bartlam, Gerlof Sebastiaan Winkler

**Affiliations:** ^1^ State Key Laboratory of Medicinal Chemical Biology, College of Life Sciences, Nankai International Advanced Research Institute (Shenzhen Futian), Nankai University, Tianjin, China; ^2^ School of Pharmacy, University of Nottingham, University Park, Nottingham, United Kingdom

**Keywords:** RNA, poly(A), gene expression, RNA degradation pathway, RNA decay, nuclease, deadenylase complex

## Abstract

In eukaryotic cells, the synthesis, processing, and degradation of mRNA are important processes required for the accurate execution of gene expression programmes. Fully processed cytoplasmic mRNA is characterised by the presence of a 5′cap structure and 3′poly(A) tail. These elements promote translation and prevent non-specific degradation. Degradation via the deadenylation-dependent 5′-3′ degradation pathway can be induced by trans-acting factors binding the mRNA, such as RNA-binding proteins recognising sequence elements and the miRNA-induced repression complex. These factors recruit the core mRNA degradation machinery that carries out the following steps: i) shortening of the poly(A) tail by the Ccr4-Not and Pan2-Pan3 poly (A)-specific nucleases (deadenylases); ii) removal of the 5′cap structure by the Dcp1-Dcp2 decapping complex that is recruited by the Lsm1-7-Pat1 complex; and iii) degradation of the mRNA body by the 5′-3′ exoribonuclease Xrn1. In this review, the biochemical function of the nucleases and accessory proteins involved in deadenylation-dependent mRNA degradation will be reviewed with a particular focus on structural aspects of the proteins and enzymes involved.

## Introduction

In eukaryotic cells, mature mRNAs transported from the nucleus to the cytoplasm contain a 3′poly(A) tail and a 7-methylguanylate (m^7^G) cap structure at the 5′end (Wilusz et al., 2001; Vicens et al., 2018; Passmore and Coller, 2022). These features are present on virtually all mRNAs with the notable exception of the histone coding mRNAs, which are characterised by the absence of a poly(A) tail. The poly(A) tail prevents non-specific degradation by 3′-5′ exoribonucleases, while the m^7^G cap structure carries out a similar protective role of the 5′end of the mRNA. In addition to their role in preventing premature degradation, both modifications are also required for efficient translation (Wilusz et al., 2001; Vicens et al., 2018; Passmore and Coller, 2022).

Cis-acting sequence elements located in the 3′untranslated region (UTR) of mRNAs can induce degradation (Raisch and Valkov, 2022; Pavanello et al., 2023). The trans-acting factors recruited by these sequence elements typically induce mRNA degradation via deadenylation coupled to the 5′-3′ degradation pathway (Muhlrad et al., 1994; Stoecklin et al., 2006; Bonisch et al., 2007). Well-characterised examples include the A/U-rich element (ARE) (Stoecklin et al., 2006), microRNA binding sites (Behm-Ansmant et al., 2006; Piao et al., 2010), and sequence elements recognised by developmental regulators such as Smaug and Bicaudal-C (Zaessinger et al., 2006; Chicoine et al., 2007; Pekovic et al., 2023). In addition to recruitment by mRNA-specific factors, the 5′-3′ degradation pathway can be induced by general mechanisms involving the interaction between the cytoplasmic poly(A)-binding protein (PABPC) 1 and members of the BTG/Tob family of proteins, which include TOB1 and its paralogue TOB2, and the BTG1 and BTG2 paralogues that are frequently mutated in non-Hodgkin lymphoma (Mauxion et al., 2009; Winkler, 2010; Yuniati et al., 2018; Almasmoum et al., 2021).

The components involved in the deadenylation-dependent 5′-3′ degradation pathway are conserved, and detailed insight into the role of the core components has become available through a large number of studies investigating the structure of the enzymes and proteins involved from various model organisms, including *Saccharomyces cerevisiae*, *Schizosaccharomyes pombe*, *Kluyveromyces lactis*, *Drosophila melanogaster*, and *Homo sapiens*. Here, we will review the structure and function of the core components in deadenylation-dependent 5′-3′ degradation and focus on the wealth of structural insight obtained in the past decade.

## Overview of the deadenylation-dependent 5′-3′ degradation pathway

**FIGURE 1:**
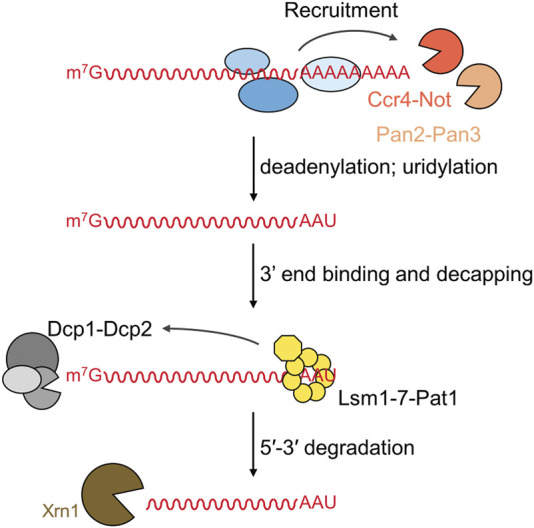
Overview of the deadenylation-dependent 5′-3′ mRNA degradation pathway. The 5′-3′ degradation pathway involves the following sequential steps: (i) deadenylation by the Pan2-Pan3 and Ccr4-Not complexes; (ii) uridylation of oligoadenylated RNA; (iii) 3′end binding by the Lsm1-7-Pat1 complex followed by recruitment of and decapping by the Dcp1-Dcp2 complex; and (iv) degradation of the mRNA by the 5′-3′ exoribonuclease Xrn1.

The 5′-3′ mRNA degradation involves distinct steps that are completed consecutively (Parker and Song, 2004; Jonas and Izaurralde, 2015) (Figure 1). The first phase in the 5′-3′ degradation pathway is the shortening of the poly(A) tail (deadenylation) (Raisch and Valkov, 2022; Pavanello et al., 2023). This step is carried out by two multi-subunit nuclease complexes that selectively recognise poly(A) RNA. While the Pan2-Pan3 deadenylase may primarily be involved in general deadenylation, degradation of a target mRNA appears to be mainly regulated by recruitment of the Ccr4-Not complex by factors binding in the 3′UTR of the messenger (reviewed in Pavanello et al., 2018; Raisch and Valkov, 2022; Wahle and Winkler, 2013). Following deadenylation, the Lsm1-7-Pat1 complex binds at the 3′end of the mRNA and recruits the Dcp1-Dcp2 decapping complex, which removes the m^7^G cap from the 5′end (Parker and Song, 2004; Jonas and Izaurralde, 2015). In *S. pombe* and mammalian cells, uridylation of degradation intermediates takes place after deadenylation, but prior to binding of the Lsm1-7-Pat1 complex (Rissland and Norbury, 2009; Scott and Norbury, 2013; Lim et al., 2014). In the final stage of the 5′-3′ degradation pathway, the mRNA body is degraded by the conserved 5′-3′ exonuclease Xrn1 (Parker and Song, 2004; Nagarajan et al., 2013).

## Deadenylation: Shortening of the poly (A) tail

The main deadenylases implicated in the 5′-3′ degradation pathway are the Pan2-Pan3 complex and the multi-subunit Ccr4-Not deadenylase (Boeck et al., 1996; Brown et al., 1996; Tucker et al., 2001; Yamashita et al., 2005; Wahle and Winkler, 2013). Pan2-Pan3 has an intrinsic affinity for cytoplasmic poly(A)-binding protein (PABPC) and prefers long poly(A)-PABPC ribonucleoprotein particles (RNPs). In addition, the GW182 (TNRC6) component of the miRNA repression complex has been implicated in mRNA-specific recruitment of Pan2-Pan3 (Braun et al., 2011; Fabian et al., 2011). By contrast, Ccr4-Not has no direct affinity for PABPC. Instead, it can interact with member so the BTG/Tob family of proteins in metazoan organisms (Mauxion et al., 2009; Winkler, 2010). The BTG1/BTG2 and TOB1/TOB2 components of this family have been shown to interact with PABPC and Ccr4-Not, thereby stimulating deadenylation of poly(A)-PABPC RNPs (Ezzeddine et al., 2007; Stupfler et al., 2016). Ccr4-Not appears to be the dominant deadenylase recruited to target mRNAs as a large number of interactions with RNA-binding proteins have been established, and the mode of recruitment has been determined in several cases in molecular detail (Raisch and Valkov, 2022; Pavanello et al., 2023).

## Deadenylation: the Pan2-Pan3 complex

The Pan2-Pan3 deadenylase is one of two deadenylase complexes implicated in the initiation of the 5’-3’ decay of mRNA by Xrn1. The Pan2-Pan3 complex is highly conserved (Uchida et al., 2004; Wahle and Winkler, 2013) with an atypical architecture in which an asymmetric Pan3 homodimer is bound by a single Pan2 subunit (Pan2:Pan3 = 1:2) (Jonas et al., 2014; Schafer et al., 2014; Wolf et al., 2014).

### The catalytic subunit Pan2

**FIGURE 2:**
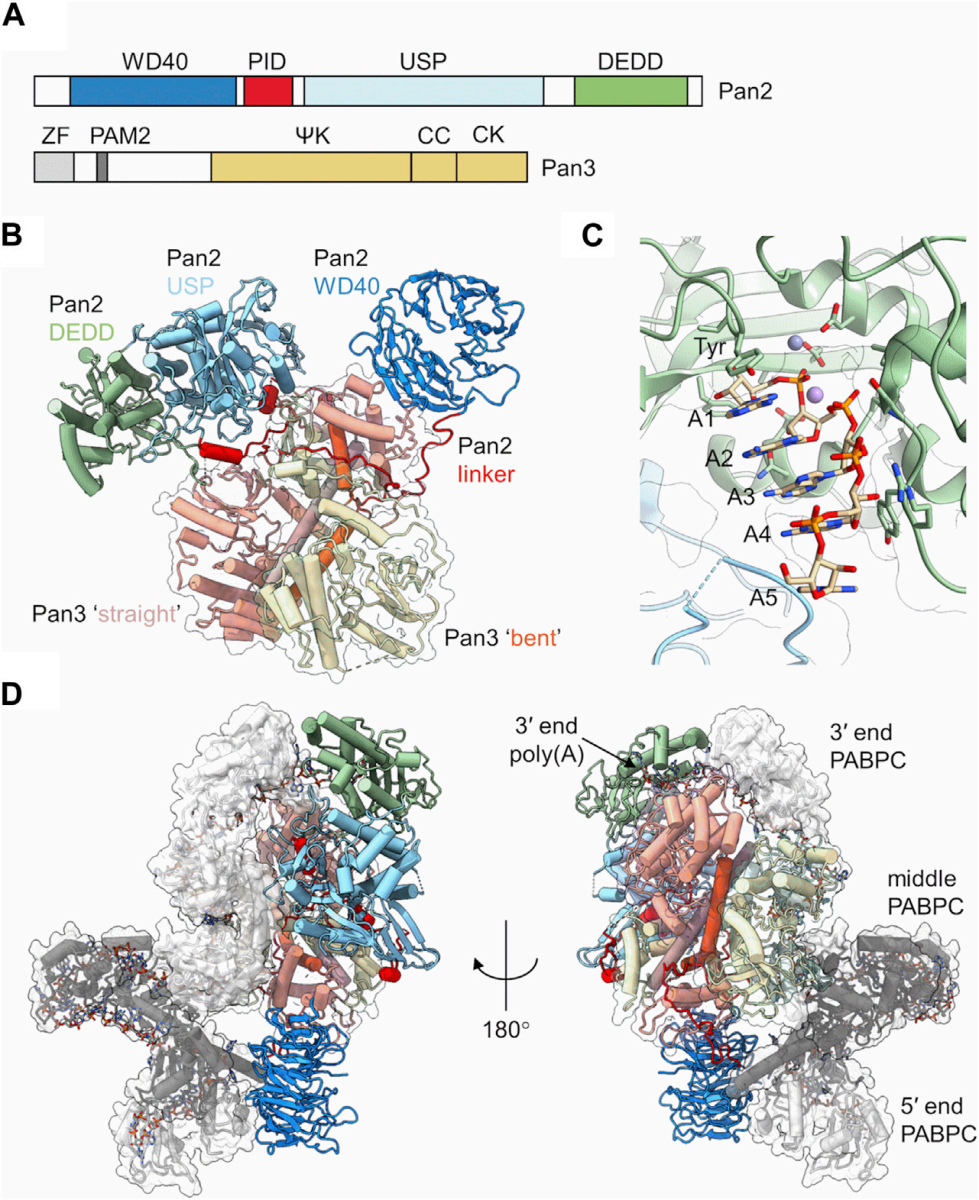
The Pan2-Pan3 deadenylase. **(A)** Schematic diagram of the domain organisation of Pan2 and Pan3. PID, Pan3 interacting domain; USP, ubiquitin-specific protease; DEDD, Asp-Glu-Asp-Asp catalytic domain. ZF, zinc finger; ΨK, pseudo-kinase; CC, coiled-coil; CK, C-terminal knob. **(B)** Overview of the *S. cerevisiae* Pan2-(Pan3)_2_ complex. Indicated are the two Pan3 protomers (tan, yellow) with the long α-helices of the coiled coil highlighted (orange, brown). Also indicated are the domains of Pan2: WD40 (blue), PID linker domain (red), USP (light blue), and catalytic DEDD domain (green). PDB entry: 6R5K (Schafer et al., 2019). **(C)** Poly **(A)** recognition by the Pan2 catalytic domain. Indicated are A5 form A helical RNA stacked onto a conserved tyrosine. PDB entry: 6R9J (Tang et al., 2019). Also indicated are two metal ions obtained by superimposition of the *S. pombe* Pop2 enzyme. PDB entry: 3G0Z (Andersen et al., 2009). **(D)** Recognition of poly**(A)**-PABPC ribonucleoprotein particles by *S. cerevisiae* Pan2-Pan3. Indicated are the domains of Pan2 and Pan3 as shown in panel **(B)**. In addition, three PABPC proteins are indicated (grey; dark grey; white). The RNA strand is shown using a stick model. PDB entry: 6R5K (Schafer et al., 2019).

Pan2 is the large subunit of the complex (Figure 2A). It contains a C-terminal exoribonuclease that belongs to the DEDD family of exonucleases. It is Mg^2+^ dependent and releases 5′-AMP upon hydrolysis of the poly(A) mRNA tail using a two-metal catalytic mechanism (Uchida et al., 2004; Wahle and Winkler, 2013; Tang et al., 2019). Pan2 displays low affinity for RNA, and has modest catalytic activity in absence of Pan3 (Schafer et al., 2014; Wolf et al., 2014). By contrast, Pan2 shows readily detectable deadenylation activity and specificity for poly(A) upon binding of its complex partner Pan3 (Wolf et al., 2014). In addition to its catalytic domain, Pan2 contains three further conserved regions (Uchida et al., 2004; Wahle and Winkler, 2013; Jonas et al., 2014; Schafer et al., 2014; Wolf et al., 2014). At the N-terminus, a WD40 domain is located, which forms a typical seven-blade β-propeller that mediates interactions with Pan3 (Jonas et al., 2014; Schafer et al., 2014) (Figure 2B). Together with the WD40 domain, a Pan3-Interacting Domain (PID) linker region adjacent to the WD40 domain is also required for complex formation with Pan3 (Jonas et al., 2014; Schafer et al., 2014; Wolf et al., 2014). This sequence contains several conserved residues responsible for binding the coiled coil regions of the Pan3 homodimer (Figure 2B). Upon binding the Pan3 homodimer, the linker sterically prevents a second WD40 domain from binding resulting in a stable complex composed of a single Pan2 subunit, and two Pan3 protomer (Jonas et al., 2014; Schafer et al., 2014). Located between the linker region and the catalytic DEDD domain is a ubiquitin-specific protease (USP) domain. This domain, however, lacks residues in the catalytic triad that are essential for activity, and the domain shows no protease activity (Quesada et al., 2004). Instead, the Pan2 USP domain engages in extensive interactions with the Pan2 C-terminal exonuclease domain (Jonas et al., 2014; Schafer et al., 2014; Wolf et al., 2014) (Figure 2B).

### The regulatory subunit Pan3

Pan3 forms an asymmetric homodimer when assembled into the yeast Pan2-Pan3 complex (Christie et al., 2013; Jonas et al., 2014; Schafer et al., 2014; Wolf et al., 2014). It displays five conserved regions (Figure 2A). At the N-terminus, a CCCH-type zinc-finger domain is located that has preference for poly(A) binding (Wolf et al., 2014). In addition, a short PAM2 motif is present, which can associate with the C-terminal MLLE domain of cytoplasmic poly(A)-binding protein (Uchida et al., 2004; Siddiqui et al., 2007). The central part of Pan3 contains a pseudokinase (ΨK) domain. The ΨK domain lacks catalytic residues required for kinase activity, but has retained the ability to bind ATP in a Mg^2+^-dependent manner (Christie et al., 2013). Moreover, the ability for nucleotide binding seems required for the ribonuclease activity of the Pan2-Pan3 complex (Christie et al., 2013). A coiled-coil (CC) region connects the ΨK domain and the C-terminal knob (CK) domain in the C-terminus of the protein. The Pan3 homodimer is formed by extensive interactions between the ΨK, CC and CK domains of one Pan3 protomer with the corresponding regions in the second protomer (Christie et al., 2013; Jonas et al., 2014; Schafer et al., 2014; Wolf et al., 2014) (Figure 2B). The resulting homodimer is asymmetric, and a notable difference can be seen in the CC regions of the two Pan3 protomers. In one protomer, the CC region forms a long ‘straight’ α-helix, whereas the CC region of the second protomer has a pronounced bend (Figure 2B). The Pan3 homodimer binds a single Pan2 subunit via the N-terminal WD40 domain and the PID linker of Pan2 (Jonas et al., 2014; Schafer et al., 2014; Wolf et al., 2014). The WD40 domain of Pan2 binds laterally to the CK domain of the Pan3 protomer containing the CC in the ‘bent’ orientation. The PID linker of Pan2 wraps around the CC regions and interacts with the CK domain of the protomer containing the ‘straight’ CC conformation thereby preventing the association of the WD40 domain of a second Pan2 protomer (Jonas et al., 2014; Schafer et al., 2014).

### Recognition and deadenylation of poly(A) ribonucleoprotein particles

The catalytic domain of Pan 2 can accommodate up to seven adenosines in the active site (Tang et al., 2019). Intriguingly, the poly(A) substrate is not recognised by selective interactions with the nucleobases. Instead, extensive hydrogen bonding takes place between Pan2 residues and the phosphate-sugar backbone of the poly(A) substrate (Tang et al., 2019) (Figure 2C). Specificity of substrate recognition is based on the intrinsic ability of poly(A) RNA to adopt an A form single stranded helical RNA conformation that depends on multiple base-base stacking interactions within the poly(A) sequence (Figure 2C). The 5′terminal adenosine stacks onto a conserved tyrosine in the active site, which positions the scissile bond towards the metal ions resulting in release of AMP (Figure 2C). The presence of guanosine residues, which disrupt the helical A-form RNA structure, interfere with productive nucleolytic activity, while cytosine and uracil residues, which allow the formation of A-form RNA, are permitted (Tang et al., 2019).

Whereas the N-terminal zinc-finger domain of Pan3 contributes to RNA binding and specificity for poly(A), removal of this domain does not significantly impair *in vitro* deadenylase activity (Wolf et al., 2014; Schafer et al., 2019). However, even though Pan2-Pan3 nuclease activity is stimulated by the presence of PABPC, removal of the PAM2 motif of Pan3 has moderate effect on the activity of the Pan2-Pan3 complex when short oligo(A) substrates are used *in vitro* (Wolf et al., 2014; Schafer et al., 2019). This is in marked contrast to the increased length of poly(A) tails in yeast containing inactivating point mutations in this region of Pan2 (Mangus et al., 2004). However, nuclease activity of Pan2-Pan3 is markedly increased in the presence of long poly(A) substrates containing 70 or 90 nucleotides that can accommodate two or three PABPC subunits (Schafer et al., 2019). The Pan2-Pan3 complex recognises a RNP containing a 90-mer poly(A) bound to three PABPC molecules through two main interactions (Figure 2D). First, Pan2 binds via the wider base of the WD40 domain to the RRM1 domain of the PABPC protein located at the 5′end of the 90-mer substrate. Secondly, RRM1 and RRM2 of PABPC located at the 3′end of the poly(A) tail interact with the USP and catalytic domains of Pan2, and position the 3′residues of the substrate into the active site of Pan2 thereby providing a rationale for enhanced deadenylation of poly(A) substrates containing multiple PABPC proteins (Schafer et al., 2019).

## Deadenylation: the Ccr4-Not complex

The Ccr4-Not (carbon catabolite repression–negative on TATA-less) complex is the main deadenylase linked to initiation of mRNA degradation via the 5’-3’ pathway. A number of regulators of mRNA stability have been shown to directly interact with the complex thereby initiating shortening of the poly(A) tail of the mRNA target and initiation of the degradation pathway (recently reviewed by Pavanello et al., 2023; Raisch and Valkov, 2022).

**FIGURE 3:**
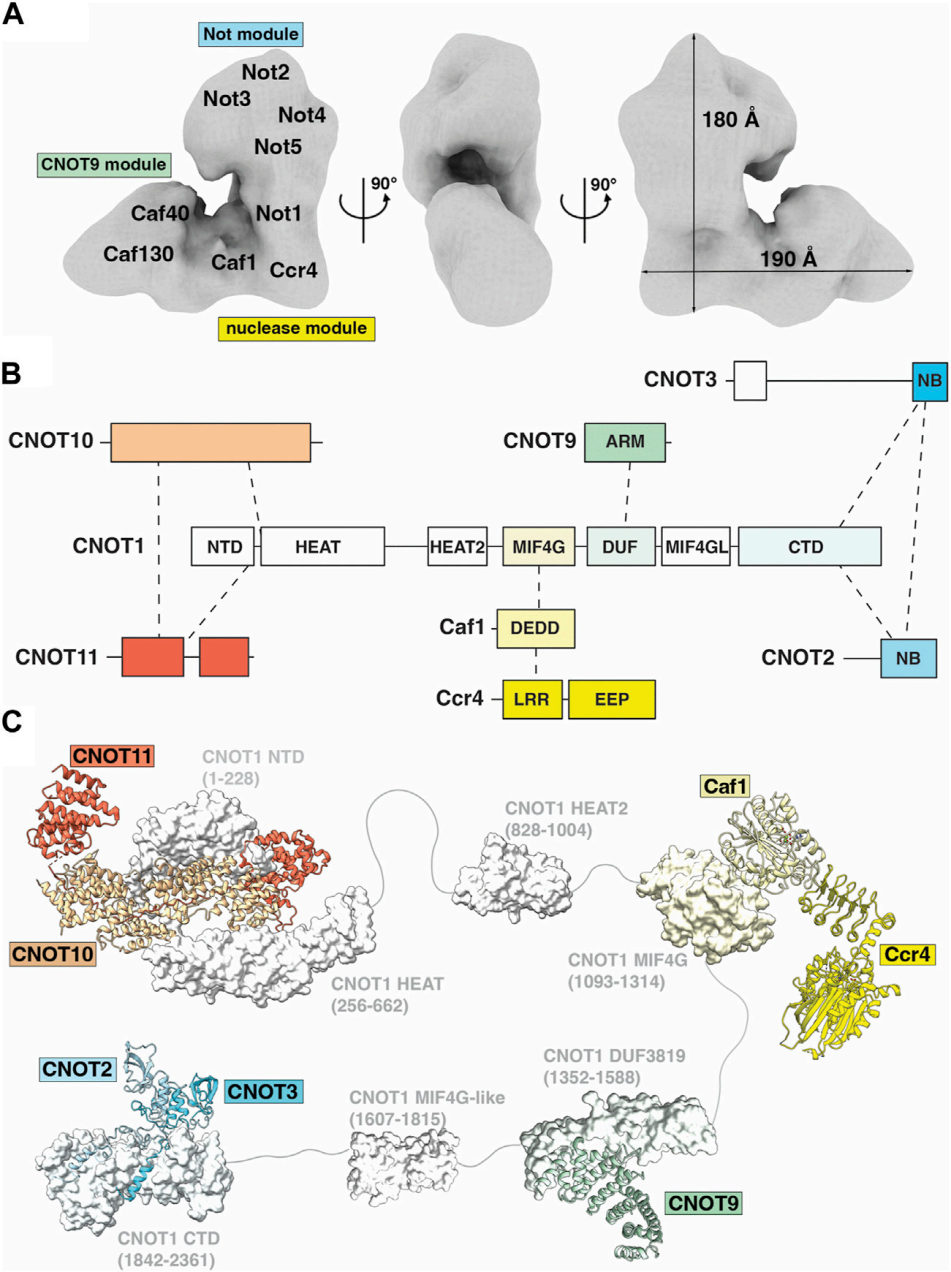
The Ccr4-Not deadenylase. **(A)** Surface representation of the three-dimensional ‘L-shaped’ map from electron microscopic analysis of the *S. cerevisiae* Ccr4-Not complex. EMDB: EMD-1901 (Nasertorabi et al., 2011). Tentative positions of subunits and modules are labelled and approximate dimensions of the complex are given. **(B)** Overview of the Ccr4-Not complex subunit architecture. **(C)** Available structures for the Ccr4-Not complex. Indicated are the N-terminal module composed of the N-terminal region of CNOT1, CNOT10 (light orange) and CNOT11 (dark orange), PDB entry: 8BFI (Mauxion et al., 2023); MIF4G-like domain 1 of CNOT1, PDB entry: 4J8S (Fabian et al., 2013); the nuclease module composed of the CNOT1 MIF4G domain, Caf1/CNOT7 (light yellow) and Ccr4/CNOT6L (dark yellow), PDB entries 3NGQ and 7VOI (Wang et al., 2010; Zhang et al., 2022); the CNOT9 module, PDB entries 4CT6 or 4CRV (Chen et al., 2014; Mathys et al., 2014) composed of the DUF3819 domain of CNOT1 and CNOT9 (green); a second MIF4G-like domain of CNOT1 modelled using AlphaFold (Jumper et al., 2021); and the NOT module composed of the CNOT1 C-terminal domain and the conserved NOT-Box regions located at the C-termini of CNOT2 (light blue) and CNOT3 (blue), PDB entry: 4C0D (Boland et al., 2013). Colours correspond to subunits in panel **(B)**. Numbers in brackets refer to the amino acid residues of CNOT1. Modules are connected by grey linkers to indicate the flexibility of the complex. **(B,C)** are adapted from Figure 2, Pavanello et al. (2023), used under CC BY 4.0.

Ccr4-Not is a large, multi-subunit protein complex of approximately 675 kDa, with a minimum of five ‘canonical’ subunits in the human complex; CNOT1, CNOT2, CNOT3/5, Ccr4 and Caf1/Pop2 (Collart and Panasenko, 2012; Wahle and Winkler, 2013). The first structural view of the Ccr4-Not complex was revealed by electron microscopy analysis of single particles of the 1 MDa *S. cerevisiae* complex, consisting of nine subunits (Nasertorabi et al., 2011). The 33 Å electron microscopy map suggests that Ccr4-Not exists in a flat ‘L-shaped’ configuration with two arms of similar length connected via a central hinge domain (Figure 3A) (Nasertorabi et al., 2011). At the time of writing, high resolution structural information for the complete Ccr4-Not complex has so far proven elusive (Ukleja et al., 2016; Raisch et al., 2019), most likely due to intrinsic flexibility of the complex.

The largest constituent component of human Ccr4-Not is CNOT1, a 2,376 amino acid protein that contains at least six structured domains identified to date (Figure 3B). CNOT1 serves as a molecular scaffold to provide binding sites for the other Ccr4-Not subunits (Figure 3B). Structural information is currently available for four ‘modules’ in Ccr4-Not: the ‘N-terminal module’ encompassing the NTD (N-terminal domain) and HEAT domains of CNOT1 and the CNOT10-CNOT11 heterodimer; the ‘Nuclease module’ consisting of the CNOT1 MIF4G domain and the catalytic subunits Caf1 and Ccr4; the ‘CNOT9 module’ formed by the CNOT1 DUF3819 (CN9BD) domain and CNOT9; and the ‘NOT module’ composed of the CNOT1 CTD (C-terminal domain) and the CNOT2-CNOT3 heterodimer (Figures 3B, C). The roles of these modules will be discussed in greater detail below. An additional MIF4G domain C-terminal to the ‘CNOT9 module’ has been identified but no function has been assigned. Linkers connecting the structured CNOT1 domains provide it with a degree of flexibility. Experimental depletion of CNOT1 both reduces the amount of other Ccr4-Not subunits and suppresses the formation of P-bodies, cellular aggregations of mRNA decay components (Ito et al., 2011). Liver-specific disruption of CNOT1 leads to increased mRNAs for transcription factors, cell cycle regulators and DNA damage response proteins due to reduced deadenylation, as well as aberrant gene expression associated with lethal hepatitis (Takahashi et al., 2020).

Key differences between yeast and vertebrate Ccr4-Not complexes are evidenced by their subunit compositions. Whereas the N-terminal region of *Drosophila* and human CNOT1 binds the CNOT10-CNOT11 heterodimer (Bawankar et al., 2013; Mauxion et al., 2013; Mauxion et al., 2023), the N-terminal region of Not1 in fungi binds Caf130 which is non-conserved in metazoans (Chen et al., 2001). The *Schizosaccharomyces pombe* Ccr4-Not complex uniquely includes the RNA-interacting subunit Mmi1 as a stable component (Ukleja et al., 2016). The E3 ubiquitin ligase Not4, a partner of the E2 conjugating enzyme UbcH5b (Ubc4/5 in yeast), is stably associated with Not1 in fungi (Collart and Panasenko, 2012; Bhaskar et al., 2015). In contrast, CNOT4 is not stably attached to metazoan Ccr4-Not complexes but interacts with CNOT9 via a short well-conserved C-terminal peptide motif (CBM) and with the NOT module (Keskeny et al., 2019). The N-terminal of CNOT4 is understood to inhibit the interaction of the CBM with Ccr4-Not and some structural reorganization is required to facilitate the interaction.

### The N-terminal module

The ‘N-terminal module’ of metazoan Ccr4-Not includes CNOT10 and CNOT11 assembled around the N-terminal region of CNOT1 (CNOT1N hereafter), as evidenced by purification of CNOT10 and CNOT11 with endogenous Ccr4-Not complexes from human and *Drosophila* cells (Bawankar et al., 2013; Mauxion et al., 2013). However, both CNOT10 and CNOT11 are considered ‘non-canonical’ subunits. CNOT1N consists of two structured domains: the NTD and HEAT domains. The first structural evidence of CNOT1N revealed HEAT repeats that are implicated in protein-protein interactions (Basquin et al., 2012). In the metazoan complex, the HEAT repeats facilitate the binding of CNOT11, in turn providing a binding surface for CNOT10, and together forming the N-terminal module (Bawankar et al., 2013). Evidence suggests that a domain of unknown function (DUF2363) in CNOT11 is responsible for tethering the protein to CNOT10, as protein fragments containing DUF2363 can bind Not10 with equal efficiency as the full-length protein (Bawankar et al., 2013). A more recent structure of the human CNOT1-CNOT10-CNOT11 complex revealed the detailed architecture of the N-terminal module (Figure 3C) (Mauxion et al., 2023). CNOT10 and CNOT11 form a heterodimer sandwiched between two helical domains of CNOT1. CNOT10 consists of 13 tetratricopeptide repeats (TPR) stacked against each other. CNOT11 comprises of three domains: a globular helical N-terminal domain (CNOT11N), an extended middle domain (CNOT11M), and a C-terminal domain previously known as DUF2363 (CNOT11C). CNOT10 wraps around CNOT11M and packs against CNOT11N, while CNOT11C extends into the solvent and is proposed to function as an ‘antenna’. The tumor suppressor/spermatogenic factor GGNBP2 was subsequently identified as an interacting partner of CNOT11C (Mauxion et al., 2023).

### The nuclease module

The central ‘Nuclease module’ of the CNOT1 scaffold includes the MIF4G domain, which provides the binding site for the first of two nucleases within the nuclease module, named Caf1 (Daugeron et al., 2001; Jonstrup et al., 2007; Wahle and Winkler, 2013). Caf1 belongs to the RNase D family of proteins with a DEDD (Aspartate-Glutamate-Aspartate-Aspartate) active site (Figure 3C). Caf1 provides a binding platform for the second nuclease, Ccr4, which belongs to the exonuclease-endonuclease-phosphatase (EEP) family of proteins (Figure 3C) (Wang et al., 2010; Wahle and Winkler, 2013). Ccr4 consists of two domains: an N-terminal leucine-rich repeat domain (LRR) to facilitate interaction with Caf1, and a C-terminal nuclease domain. Two human paralog genes of Ccr4, CNOT6/Ccr4a and CNOT6L/Ccr4b (78% identity and 88% similarity), are mutually exclusive in the Ccr4-Not complex (Lau et al., 2009; Winkler and Balacco, 2013). Moreover, the structure of CNOT7/Caf1 by Horiuchi and colleagues determined in complex with Tob revealed the basis for the interaction with the N-terminal domain of BTG/TOB proteins (Yang et al., 2008; Horiuchi et al., 2009), which link the Ccr4-Not complex to PABPC1 and stimulate deadenylation (Ezzeddine et al., 2007).

Caf1 and Ccr4 have been shown to have non-equivalent roles in cells (Aslam et al., 2009; Mittal et al., 2011; Yi et al., 2018; Mostafa et al., 2020). A differential contribution of Caf1 and Ccr4 has also been shown from biochemical studies of the purified Ccr4-Not complex or isolated nuclease sub-complexes even though some experiments indicate that both catalytic subunits are required for deadenylation (Maryati et al., 2015; Stowell et al., 2016; Raisch et al., 2019; Chen et al., 2021; Pekovic et al., 2023). Structures of CNOT6L/Ccr4 and CNOT7/Caf1 determined in isolation from the Ccr4-Not complex revealed the molecular basis for their Mg^2+^dependent activities (Horiuchi et al., 2009; Wang et al., 2010). The human CNOT6L structure revealed five conserved catalytic residues: Asn195, Glu240, Asp410, Asp489 and His529. Two bound magnesium ions were identified in the active site, and deadenylase activity was abolished by an E240A mutant or by loss of Mg^2+^ (Wang et al., 2010). The human structure of CNOT7 in complex with the antiproliferative protein Tob revealed the conserved DEDD residues: Asp40, Glu42, Asp161 and Asp230. CNOT7 was shown to require divalent metal ions for activity, with higher activity in the presence of Mn^2+^ than Mg^2+^ (Horiuchi et al., 2009). Crystallographic studies of the structural homolog *S. pombe* Pop2p (Caf1p) identified two metal sites in the active site, with a preference for Mn^2+^ and Zn^2+^ over Mg^2+^ (Jonstrup et al., 2007; Andersen et al., 2009).

**FIGURE 4:**
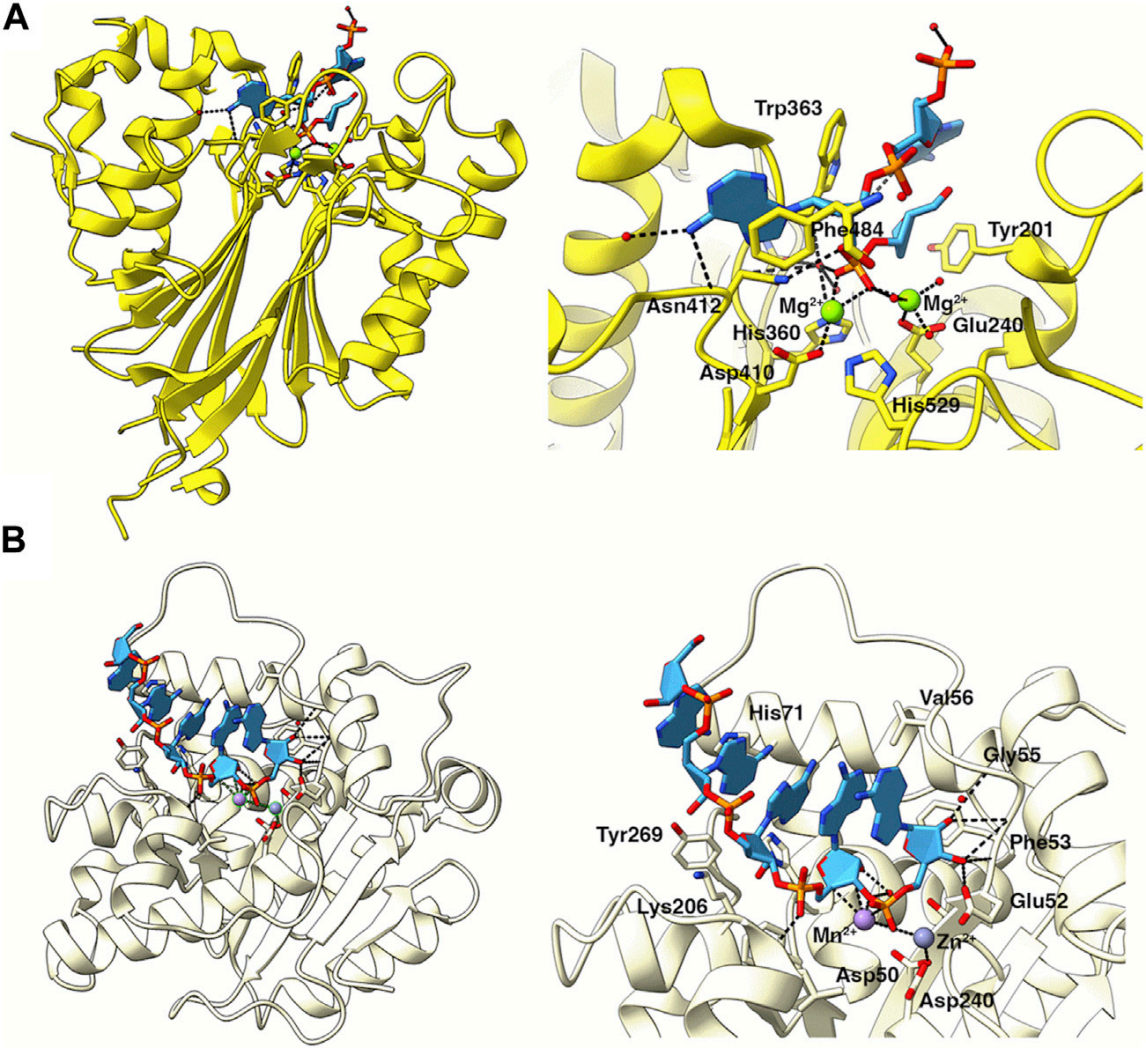
Poly **(A)** recognition by the catalytic subunits of Ccr4-Not. **(A)** Structure of the nuclease domain of human Ccr4/CNOT6L (yellow) in complex with poly **(A)** DNA (light blue). PDB entry: 3NGO (Wang et al., 2010). The right panel shows an enlarged view of poly **(A)** in the catalytic site. Key residues involved in catalysis or substrate recognition are shown as sticks as labelled. Mg^2+^ ions are shown as green spheres. Bonds are indicated by black dashed lines. **(B)** Structure of Caf1 (pale yellow), PDB entry 3G0Z (Andersen et al., 2009), with a poly **(A)** RNA substrate (light blue) modelled from the homologous Pan2 structure in complex with poly **(A)**_7_, PDB entry: 6R9J (Tang et al., 2019). The right panel shows an enlarged view of poly **(A)** in the catalytic site. Key residues involved in catalysis or substrate recognition are shown as sticks as labelled. Metal ions are shown as spheres. Bonds are indicated by black dashed lines.

Structural analysis has also revealed the different modes of poly(A) recognition by the two nucleases. Ccr4 selectively recognises poly(A) residues via specific recognition of the adenine bases (Wang et al., 2010), whereas Caf1 forms multiple interactions with the phosphate-sugar backbone with no significant base interactions (Tang et al., 2019). In CNOT6L/Ccr4, a structure with poly(A) DNA identified two complete nucleotides in the deep binding cleft. Specific base interactions with Asn412 and Phe484 explain the strict preference for adenine bases (Figure 4A) (Wang et al., 2010). While no structures of Caf1 in complex with nucleotide substrates have been determined to date, a structure of the homologous Pan2 nuclease with poly(A)_7_-RNA identified five nucleotides in the shallow binding cleft with a lack of base-specific interactions between Pan2 and adenines, suggesting Pan2 recognizes poly(A) RNA primarily through backbone interactions (Figure 4B) (Tang et al., 2019). Docking of poly(A) RNA into the Caf1 structure suggests that Caf1 recognizes poly(A) via a similar mechanism to Pan2, although poly(A) is more buried in Caf1 than in Pan2 and base-specific contacts cannot be ruled out.

Initial structural studies of the nuclease module revealed the interaction of the Ccr4 LRR domain with Caf1 but were unable to resolve the positions of the two nuclease domains relative to one another. More recent nuclease module structures have established the flexibility of the nuclease domains (Figure 3C). A human Caf1-Ccr4 (CNOT6) dimeric complex structure showed an estimated distance of 46 Å between divalent metal ions in the Caf1 and Ccr4 active sites (Chen et al., 2021), while this distance was increased to approximately 64 Å for a human CNOT1-Caf1-Ccr4 (CNOT6L) complex as evidenced by structural and electron paramagnetic resonance (EPR) spectroscopy analyses (Zhang et al., 2022). Interestingly, the active sites of Ccr4 and Caf1 are both accessible but point away when they are in complex, suggesting a spatial organisation, possibly triggered by co-factors, that would explain an apparent redundancy (Wahle and Winkler, 2013). Alternatively, allosteric regulation might facilitate the action of the two deadenylases in a cooperative fashion (Maryati et al., 2015; Pekovic et al., 2023).

### The CNOT9 module

The ‘CNOT9 module’ is composed of the CNOT1 DUF3819 domain and CNOT9. CNOT9, also known as RQCD1 (Required for Cell Differentiation 1) or Caf40, is a canonical subunit of the Ccr4-Not complex that acts as a transcriptional co-factor in embryo development, is involved in growth control and cell differentiation, and is associated with tumorigenesis (Hiroi et al., 2002; Ajiro et al., 2009; Ajiro et al., 2010; Wong et al., 2015). The DUF3819 domain, also known as CN9BD, is located immediately C-terminal to the MIF4G domain in the mammalian complex (Figure 3C). CNOT9 features a conserved ARM domain which consists almost entirely of armadillo repeats folded into a crescent shape with a positively charged cleft (Garces et al., 2007).

CNOT9 is not catalytically active, but structural evidence has shown that it is a hotspot for protein-protein interactions. The interaction of CNOT9 and CNOT1 DUF3819 reveals W-binding pockets on the convex side that can interact with specific tryptophan residues of tristetraprolin (TTP) and GW182/TNRC6 proteins (Chen et al., 2014; Mathys et al., 2014). The armadillo repeats also provide a peptide-binding pocket on the concave side that can accommodate RNA-binding proteins such as Roquin and Bag-of-marbles (Sgromo et al., 2017; Sgromo et al., 2018), as well as the conserved CBM of the E3 ubiquitin ligase CNOT4 (Keskeny et al., 2019), thus conferring an important regulatory role.

### The NOT module

The ‘NOT module’ of the Ccr4-Not complex located in the C-terminal CTD region of CNOT1 is a trimeric complex with CNOT2 (Not2) and CNOT3 (Not5) (Figure 3C). The CTD of CNOT1, while largely unstructured, contains a conserved CNOT1 superfamily homology (SH) domain (Boland et al., 2013). This SH domain provides a binding surface for CNOT2, itself tethering CNOT3 to the complex and forming the NOT domain (Raisch et al., 2018). CNOT2 and CNOT3 share similar structures at their C-terminus, which is responsible, within a larger region, for the heterodimer assembly (Bawankar et al., 2013; Boland et al., 2013). Both proteins feature a NOT1 anchoring region (NAR), a connector sequence (CS) and a NOT-box domain (Boland et al., 2013). CNOT2 and CNOT3 heterodimerise through the interaction of their NOT-box domains, while the CNOT2-CNOT3 heterodimer is tethered to CNOT1 via the NOT1 anchoring regions (Boland et al., 2013).

Structural information for the N-terminal region of CNOT2 is limited, but the N-terminus of CNOT3 (Not5) is known to form a highly conserved three-helix bundle (Buschauer et al., 2020). Cryo-electron microscopy analysis in *S. cerevisiae* showed that the Ccr4-Not complex is recruited to the ribosome via specific interaction of this Not5 N-terminal domain with the ribosomal E-site, with the requirement that the A-site is empty, tRNA is in the P-site, and the E3 ubiquitin ligase Not4 (CNOT4) is present (Buschauer et al., 2020). Binding of the CNOT3 N-terminal domain into the ribosomal E-site has been shown to be conserved in mammalian cells and requires the presence of CNOT4 (Absmeier et al., 2022).

### A model for recruitment of Ccr4-Not to mRNA via PABPC1

**FIGURE 5:**
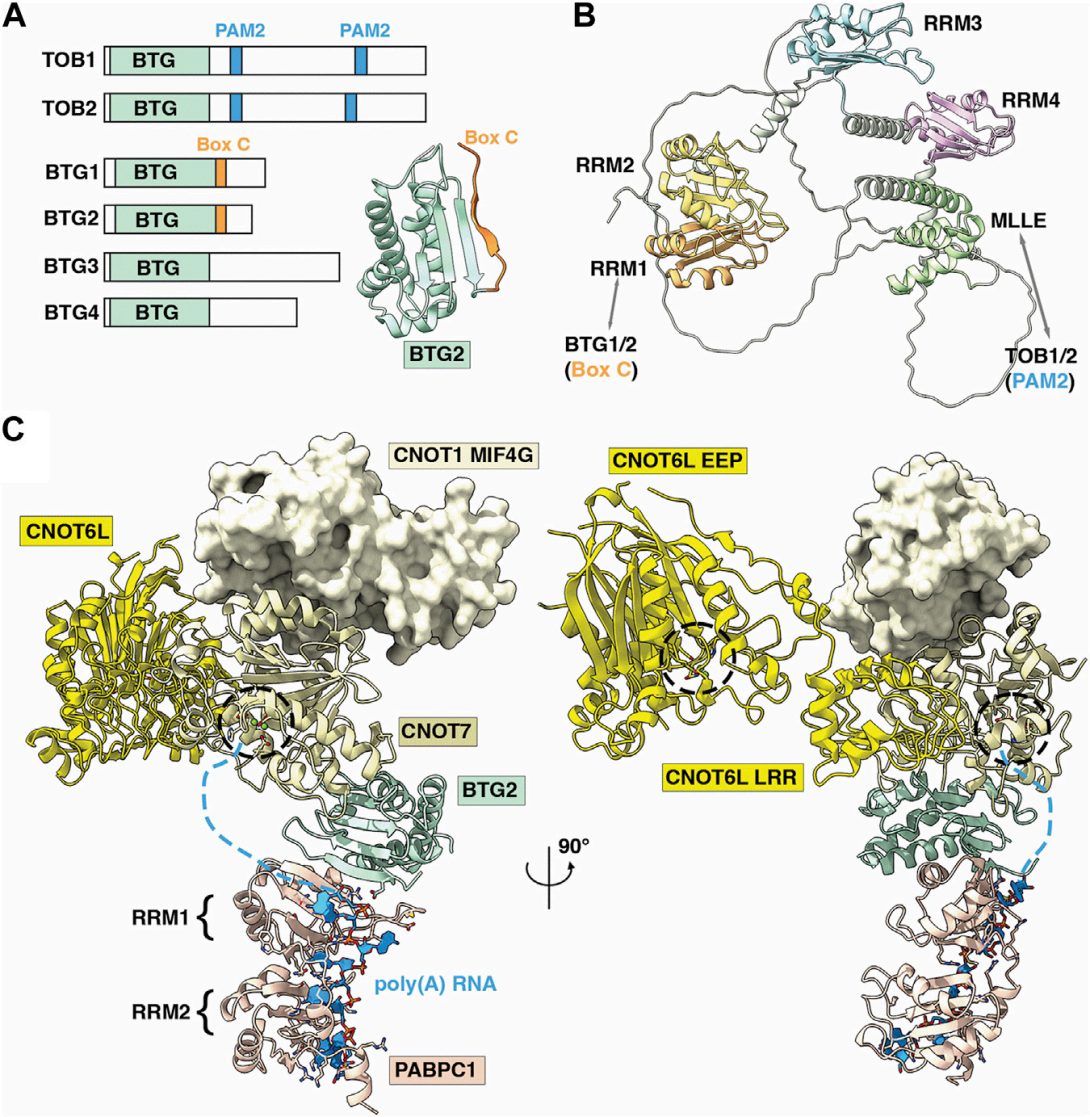
Role of the BTG-Tob proteins in deadenylation of poly **(A)**-PABPC ribonucleoprotein particles by Ccr4-Not. **(A)** Overview of BTG/TOB proteins. Shown are the conserved BTG (APRO) domain (light green), PAM2 motifs in TOB1 and TOB2 (light blue), and Box C regions in BTG1 and BTG2 (yellow). Inset is the BTG2 structure showing the APRO domain (light green) and Box C region (orange). PDB entry: 3DJU (Yang et al., 2008). **(B)** AlphaFold model of human PABPC1 (Uniprot P11940) (Jumper et al., 2021). Indicated are RRM1 (orange) that interacts with Box C regions of BTG1/BTG2, RRM2-RRM4 (yellow, light blue, pink), and the MLLE domain (green) that interacts with PAM2 motifs of TOB1/TOB2. **(C)** Model of the human nuclease module, PDB entry 7VOI (Zhang et al., 2022), in complex with BTG2-PABPC1 (RRM1-2)-poly**(A)** (PDB_Dev entry PDBDEV_00000099) (Ameerul et al., 2022). Indicated are PABPC1 (light pink); BTG2 (light green); Caf1/CNOT7 (light yellow); Ccr4/CNOT6L (yellow); CNOT1 (MIF4G domain, pale yellow) and poly**(A)** RNA (light blue). Active sites of Ccr4/CNOT6L and Caf1/CNOT7 are indicated by black dashed circles. Light blue dashed lines indicate the predicted path of poly**(A)** RNA from PABPC1 (3′ end) to the Caf1/CNOT7 active site. **(A,B)** are adapted from Figure 5, Pavanello et al. (2023), used under CC BY 4.0.

The cytoplasmic poly(A)-binding protein 1 (PABPC1) is linked to the Ccr4-Not complex via the BTG/TOB family of proteins. TOB1 and TOB2 interact with the C-terminal MLLE domain of PABPC1 via their PAM2 motifs in their extended C-termini (Ezzeddine et al., 2012) (Figures 5A, B). BTG1 and BTG2, on the other hand, interact with the first RNA recognition motif (RRM1) of PABPC1 via the short Box C motif, and both BTG2 and PABPC1 RRM are sufficient to stimulate Ccr4-Not deadenylase activity (Figures 5A, B) (Stupfler et al., 2016). TOB/BTG proteins have also been shown to interact with the Ccr4-Not subunit Caf1 via BoxA and BoxB motifs (Yang et al., 2008; Horiuchi et al., 2009). A structure of PABPC1 RRM1 and RRM2 motifs in complex with poly(A)_11_ shows that each RRM uses a β-sheet bearing highly conserved RNP1 and RNP2 sequence motifs to recognise poly(A) RNA, with the linkers between RRM domains forming a clamp to hold the RNA (Safaee et al., 2012).

The accumulation of available structures for the Ccr4-Not nuclease module, BTG2, and PABPC1 with poly(A) RNA have facilitated the construction of a model for the recruitment of Ccr4-Not to mRNA via TOB/BTG and PABPC1 (Ameerul et al., 2022). A combination of mutagenesis, NMR chemical shift perturbation and molecular docking facilitated a model for BTG2-PABPC1 in the absence of an experimental structure (Figure 5C). In the model, the 3′ end of the poly(A) RNA bound to PABPC1 is oriented towards the Caf1 active site, which degrades poly(A) RNA in a 3′-5′ manner. Thus, by serving as a bridge between Ccr4-Not (via Caf1) and PABPC1, BTG2 is able to stimulate deadenylation by the Ccr4-Not complex. A BTG2 variant lacking the ability to interact with PABPC1 does not inhibit cell cycle progression, indicating that binding to Ccr4-Not and PABPC1 is key for BTG2 function (Stupfler et al., 2016).

## Decapping: role of the Pat1-Lsm1-7 and Dcp1-Dcp2 complexes

### Binding of deadenylated RNA by the Lsm1-7-Pat1 complex

**FIGURE 6:**
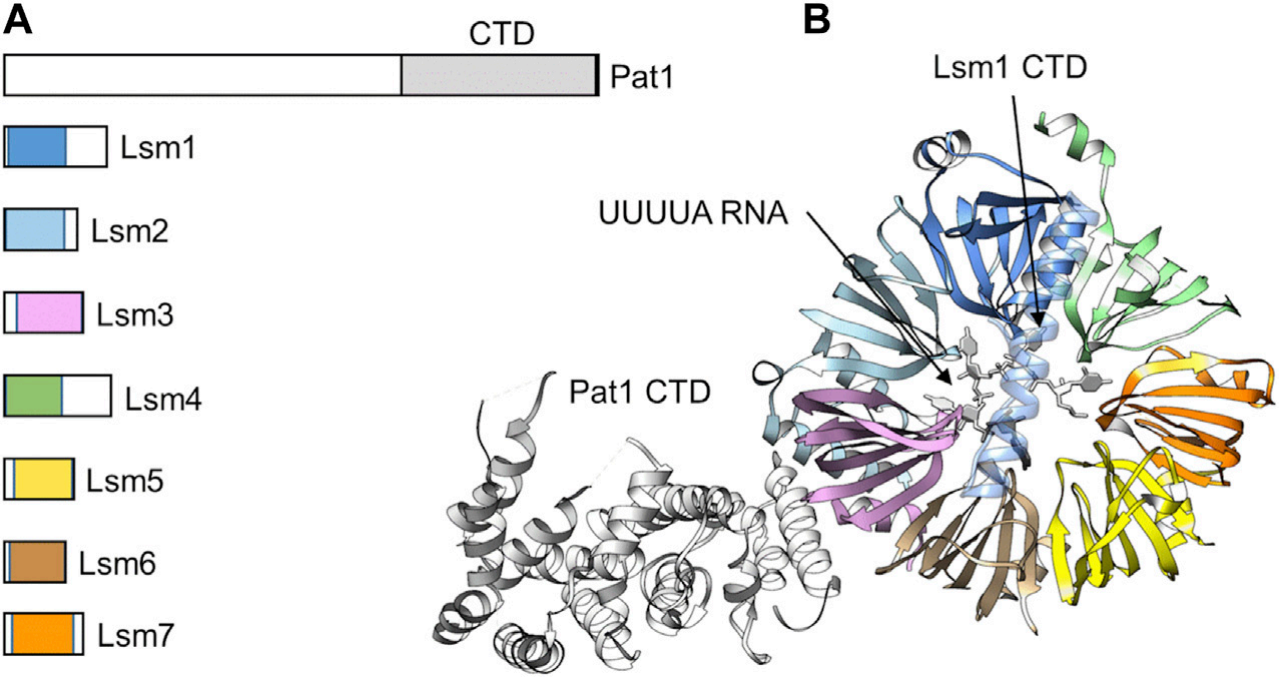
The Lsm1-7-Pat1 complex binds 3′oligoadenylated RNA. **(A)** Schematic diagram of the subunits of the Lsm1-7-Pat1 complex. **(B)** Overview of the structure of the Lsm1-7-Pat1 complex. Indicated is the β-propeller formed by Lsm1-7; the C-terminal α-helix of Lsm1; Pat1 (white) binding to Lsm2-Lsm3; and UUUA RNA (white). The model was generated by superposition of the *S. cerevisiae* Lsm1-7-Pat1 complex, PDB entry: 4C8Q (Sharif and Conti, 2013), and the *S. pombe* Lsm1-7 complex bound to UUUUA RNA, PDB entry: 6PPQ (Montemayor et al., 2020).

Following deadenylation, the oligoadenylated mRNA is bound by the Lsm1-7-Pat1 complex (Bouveret et al., 2000; Tharun et al., 2000; Haas et al., 2010). In *S. cerevisiae*, cells containing loss-of-function alleles of LSm1-7 display impaired mRNA degradation, and oligoadenylated mRNA species accumulate (Bouveret et al., 2000; Tharun et al., 2000). The oligoadenylated degradation intermediates are capped indicating that binding by the LSm1-7-Pat1 complex follows deadenylation mRNA and precedes decapping (Bouveret et al., 2000; Tharun et al., 2000). The LSm1-7-Pat complex contains seven small (MW ∼10–20 kDa) proteins that all contain a conserved Sm motif, which consists of five anti-parallel β-strands and a single α-helix, and a single Pat1 subunit (Sharif and Conti, 2013; Montemayor et al., 2020) (Figure 6A). The LSm1-7 complex is cytoplasmic and shares several subunits with the nuclear LSm2-8 complex involved in splicing. The Lsm1-7 proteins form a β-barrel with the α-helices located on one side of the heptameric ring (Sharif and Conti, 2013) (Figure 6B). A unique feature of the Lsm1 subunit is the presence of a C-terminal extension, which forms an extended αhelix that appears to occlude-the opening in the centre of the ring (Sharif and Conti, 2013; Montemayor et al., 2020). The Pat1 protein interacts with the Lsm1-7 ring via the α-helices of the Lsm2 and Lsm3 subunits and the C-terminal domain of Pat1 (Montemayor et al., 2020).

The purified Lsm1-7 complex can bind RNA with high affinity using an interface located in the centre of the Lsm1-7 ring structure (Chowdhury et al., 2014; Montemayor et al., 2020) (Figure 6B). Deletion of the C-terminal extension of Lsm1 increases the affinity of the Lsm1-7 complex for RNA suggesting a possible regulatory role for this part of the Lsm1 protein (Chowdhury et al., 2012). Lsm1-7 binds near the 3′end of RNA and specifically recognises RNA with a short oligo(A) tail (Montemayor et al., 2020). In addition, Lsm1-7-Pat1 has a strong preference of U-rich sequences near the 3′end (Montemayor et al., 2020). Addition of the Pat1 subunit increases the affinity of the complex for oligo(A) RNA via its middle and C-terminal region (Lobel et al., 2019). While the C-terminal domain of Pat1 contains a highly basic surface area with RNA-binding activity, the molecular basis of RNA recognition by Pat1 is not clear (Braun et al., 2010).

In *S. pombe* and mammalian cells, oligoadenylated mRNAs are readily uridylated (Rissland and Norbury, 2009; Lim et al., 2014). In *S. pombe*, the Cid1 enzyme is required for this activity, while TUT4 and TUT7 have been identified as the enzymes in mammalian cells (Rissland and Norbury, 2009; Lim et al., 2014). Uridylation is enhanced on oligo(A)-tailed degradation intermediates in the absence of degradation factors Lsm1, decapping factors, or Xrn1 indicating that uridylation is required for Lsm1-7-Pat1 binding and decapping. Interestingly, degradation of histone mRNA, which do not have a poly(A) tail, requires oligouridylation for decapping and degradation (Mullen and Marzluff, 2008). In addition, C/U residues with the consensus CUCU are added to deadenylated mRNA prior to decapping in the filamentous fungus *Aspergillus nidulans* (Morozov et al., 2010) suggesting that 3′end modification of the oligoadenylated degradation intermediates is a common and conserved event.

### Removal of the cap structure by the Dcp1-Dcp2 complex

**FIGURE 7:**
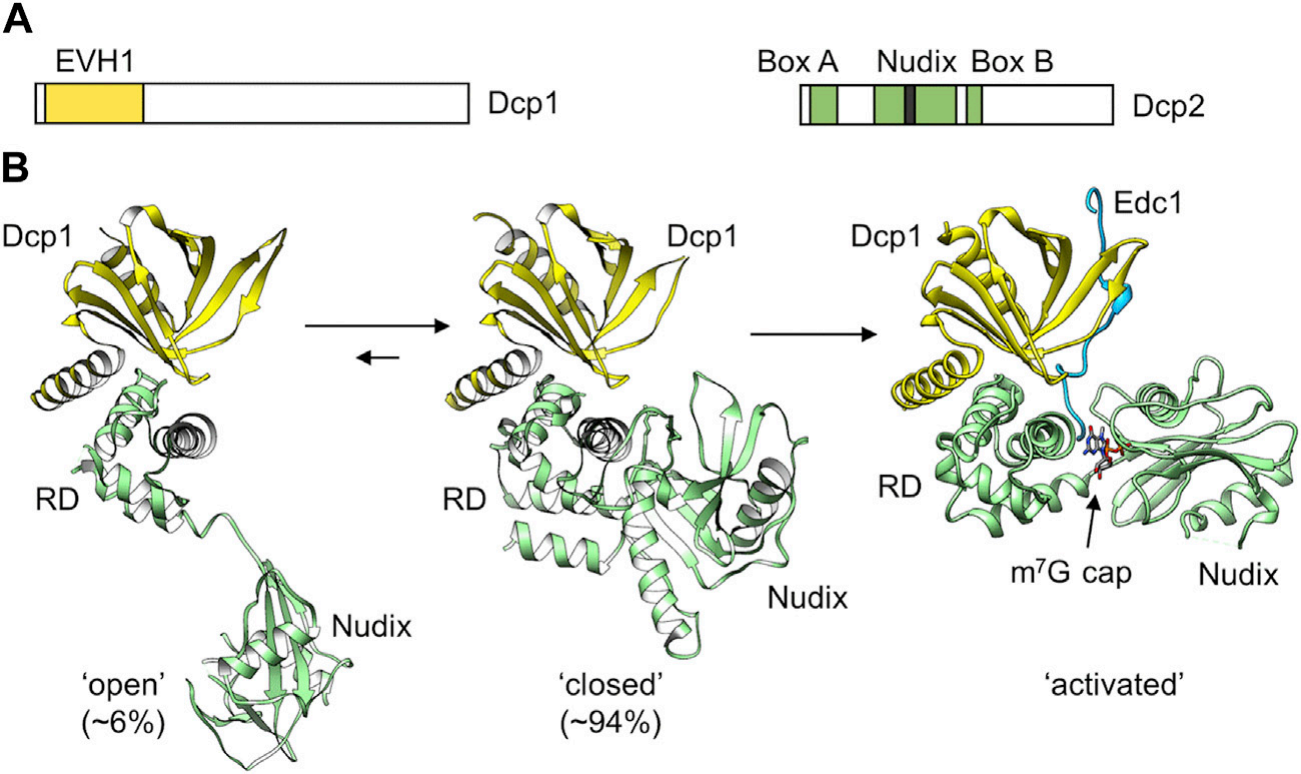
The Dcp1-Dcp2 decapping complex. **(A)** Schematic diagram of the subunits of the Dcp1 and Dcp2 proteins. Indicated are the Ena-VASP homology (EVH1) domain of Dcp1; **(B)** Structure of the Dcp1-Dcp2 complex. Indicated are the ‘open’ (*left*) and ‘closed’ (*middle*) conformations adopted by the *S. pombe* Dcp1-Dcp2 dimer in solution. PDB entry: 2KQM (She et al., 2008). Also indicated is the active conformation adopted upon binding of enhancers of decapping protein 1 and RNA (Right). PDB entry: 5N2V (Wurm et al., 2017). The percentages ‘open’ and ‘closed’ were determined by (Wurm et al., 2017).

After binding of the Lsm1-7-Pat1 complex, the oligoadenylated intermediate is prepared for decapping by the Dcp1-Dcp2 heterodimeric complex (Figure 7A). Recruitment of Dcp1-Dcp2 is mediated by the Lsm1-7-Pat1 complex by interactions between Pat1 and Dcp2 (Charenton et al., 2017; Lobel et al., 2019). The Dcp1 protein contains an N-terminal EVH1 domain and a divergent C-terminal region (Arribas-Layton et al., 2013). The EVH1 domain is a protein-protein interaction domain responsible for the interaction with Dcp2, the catalytically active subunit of the complex. Dcp2 contains an N-terminal regulatory domain, a Nudix (nucleotide diphosphate linked to an X moiety) hydrolase domain, that is characterised by a 23 amino acids consensus Nudix pyrophosphatase motif (GX_5_EX_7_RE (I/L/V)XEEXG (I/L/V)). The disordered C-terminal region contains leucine-rich helical motifs that directly interact with the C-terminal domain of Pat1 (Charenton et al., 2017; Lobel et al., 2019). Box A region, which is part of the N-terminal regulatory domain, interacts with the EVH1 domain of Dcp1, whereas Box B region located in the Nudix domain has an intrinsic ability to bind RNA, and is required for its decapping activity *in vitro* (Piccirillo et al., 2003).

Dcp2 requires divalent metal ions (Mg^2+^ or Mn^2+^) for its activity to remove the cap structure and release m^7^GDP and RNA containing a 5′monophosphate group as the products (Wang et al., 2002; Piccirillo et al., 2003). The enzyme has high specificity for capped RNA, and is unable to bind the isolated cap structure (m^7^GpppG) or unmethylated cap structures with high affinity (Piccirillo et al., 2003). The activity of Dcp2 is enhanced in the presence Dcp1 (She et al., 2008). However, full activity requires the binding of enhancers of decapping, such as Edc1-4, which are disordered proteins that bind via proline-rich sequences to the regulatory domain of Dcp1 (Charenton et al., 2016; Valkov et al., 2017; Wurm et al., 2017; Mugridge et al., 2018).

The Dcp2 catalytic subunit can adopt different conformations (She et al., 2008) (Figure 7B). A flexible hinge between the N-terminal regulatory domain and the catalytic Nudix domain allows major changes in the orientation of the two domains and provides a regulatory mechanism for mRNA decapping. In a more compact, closed conformation, the N-terminal regulatory domain packs close to the catalytic domain (Figure 7B). This orientation is catalytically inactive, because the cap binding site is separated from the Nudix helix that is required for catalysis (Charenton et al., 2016; Wurm et al., 2017; Mugridge et al., 2018). Moreover, the RNA binding path (Box B) on the catalytic domain is not accessible in the closed conformation. Dcp2 can also adopt an extended conformation where the regulatory domain is distant to the catalytic domain (Figure 7B). However, in the presence of Dcp1, the closed, catalytically inactive conformation is predominantly induced (Wurm et al., 2017). While binding of the enhancer of decapping Edc1 alone does not influence the conformation of the Dcp1-Dcp2 complex, a major conformational change occurs upon binding of capped RNA in the presence of Edc1. In the active conformation, Edc1 interacts with Dcp1 and both the regulatory and catalytic domains of Dcp2 (Figure 7B). The catalytic site is composed of residues from both the N-terminal regulatory and Nudix domain. For instance, the terminal methylated guanosine residue stacks with a conserved tryptophan of the regulatory domain, while 3 Mg^2+^ ions required for catalysis are coordinated by residues of the Nudix domain. In addition, an a positively charged putative RNA binding channel that includes Box B residues of Dcp2 extends from the catalytic site (Charenton et al., 2016; Wurm et al., 2017; Mugridge et al., 2018).

## Degradation: the Xrn1 nuclease

**FIGURE 8:**
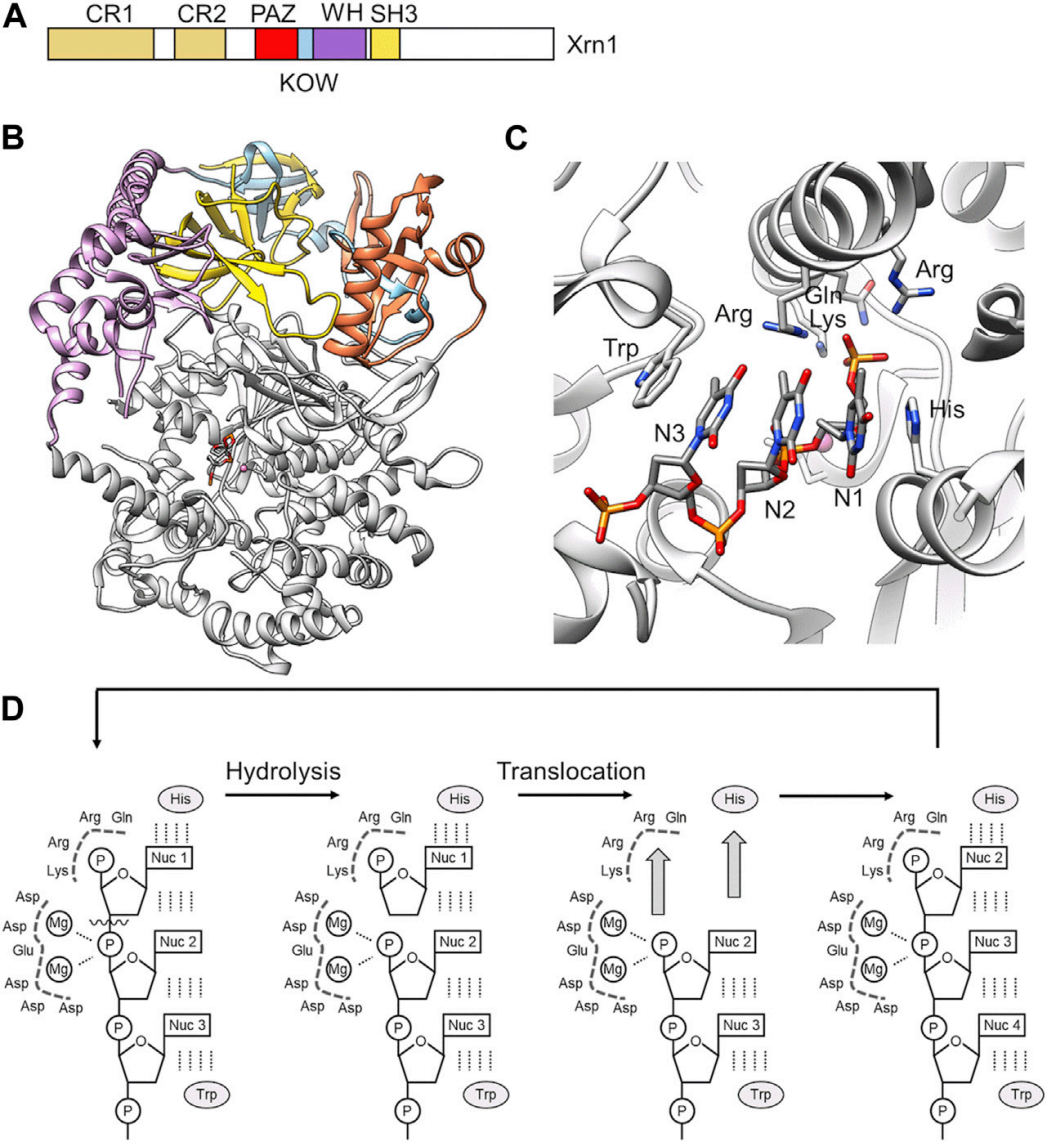
RNA degradation by the 5′-3′ exoribonuclease Xrn1. **(A)** Schematic diagram and domain organisation of the Xrn1 nuclease. Indicated are the following regions: CR1, conserved region 1; CR2 conserved region 2; PAZ, Piwi Argonaut and Zwille; KOW, Kyrpides, Ouzounis and Woese; WH, winged helix; SH3, SH3-like domain. The C-terminal region (white) is disordered. **(B)** Overview of the structure of the structured N-terminal region of *D. melanogaster* Xrn1. PDB entry: 2Y35 (Jinek et al., 2011). **(C)** The active site of Xrn1 containing a three-nucleotide DNA substrate. Indicated are residues of the basic pocket (Arg, Lys, Gln, Arg) and the His and Trp residues stacking the nucleotides in the active site. **(D)** Proposed processive mechanism of catalysis by Xrn1 (Jinek et al., 2011).

In the final step, the decapped mRNA is degraded in the 5′-3′ direction by the Xrn1 nuclease generating 5′monophosphate nucleotides (Stevens, 1980; Nagarajan et al., 2013). In the absence of Xrn1, partially decapped mRNA species lacking a poly(A) tail accumulate (Hsu and Stevens, 1993). Exonucleolytic degradation is linked to decapping by direct interactions between Xrn1 and the Dcp1-Dcp2-Edc4 complex (Braun et al., 2012). Xrn1 is a highly conserved, high molecular weight, single polypeptide exoribonuclease with processive activity (Stevens, 2001) (Figure 8A). Xrn1 can also degrade DNA although at a slower rate compared to its preferred RNA substrate (Stevens, 2001). Enzyme activity requires the presence of divalent metal ions (Stevens, 1980), which are coordinated by seven, highly conserved acidic residues (Jinek et al., 2011).

In addition to the conserved nuclease domain, which is located at the N-terminus of Xrn1, and conserved PAZ, KOW, Winged helix, and SH3-like regions in the middle part of the protein, Xrn1 contains an extensive disordered C-terminal region (Figure 8A). The crystal structures of the conserved regions of *D. melanogaster* and *K. lactis* Xrn1 provide insight into the overall organisation of the conserved domains as well as substrate recognition (Chang et al., 2011; Jinek et al., 2011). The conserved regions encompassing the catalytic domain of Xrn1 form a largely α-helical, globular conformation with the catalytic site located in its centre (Figure 8B). The conserved PAZ, KOW, Winged helix and SH3-like regions are stacked on top of the globular, catalytic domain. These regions likely contribute to the stability of the globular assembly. In budding yeast, the SH3-like domain provides an essential function of the protein, as the severe growth defect observed in Xrn1Δ cells cannot be rescued by expression of an Xrn1 variant lacking this region (Page et al., 1998). The Winged helix domain extends towards the catalytic centre and may have a role in regulating the activity of Xrn1 (Jinek et al., 2011).

A crystal structure of Dm Xrn1 lacking the disordered C-terminal region is available (Figure 8A). This model also contains a 5′phosphorylated 11-mer oligo (dT) DNA oligonucleotide and provides insight into substrate recognition. In this structure, a highly conserved Asp residue involved in the coordination of a Mg^2+^ ion was substituted with an Ala residue to prevent degradation of the substrate. In the model, Xrn1 only recognises the 5′terminal three nucleotides (Figure 8C), which is consistent with RNA protection analysis that indicate only a short chain of nucleotides are bound by the Xrn1 enzyme (Jinek et al., 2011). The backbone of the three nucleotides form electrostatic interactions with a positively charged surface area, while the nucleobases are stacked between an invariant His and Trp residue (Figure 8C). This binding mode indicates the absence of specific nucleobase interactions, and is consistent with the absence of a sequence preference by Xrn1. The 5′monophosphate group is specifically recognised in a basic pocket containing strictly conserved residues (Figure 8C). The pocket cannot accommodate larger 5′modifications thereby explaining the specific recognition of uncapped RNA. Moreover, the 5′phosphate group makes a critical contribution to substrate binding, because RNA lacking a phosphate at the 5′end are poor substrates.

Substrate binding positions the phosphate ester in close proximity of 2 Mg^2+^ ions. Based on similarities to the FEN-1 and T4 RNase H nucleases (Hwang et al., 1998; Mueser et al., 1996), the latter Mg^2+^ ion may activate a water molecule for nucleophilic attack at the scissile phosphate bond linking the first and second nucleotide (Jinek et al., 2011). Xrn1 is a highly processive enzyme with no partially degraded intermediates observed (Stevens, 2001). In addition to substrate recognition, the invariant His that forms π-π interactions with the 5′nucleotide and the basic pocket binding the 5′phosphate are also required for processivity as demonstrated by the analysis of alanine substitutions. Thus, Jinek et al. (Jinek et al., 2011) proposed a mechanism in which two key interactions drive translocation (Figure 8D). First, π-π stacking between the 5′nucleobase and the invariant His residue, and secondly, interactions between the 5′phosphate and the basic binding pocket (Figure 8D).

## Coordination of events in the deadenylation-dependent 5′-3′ degradation pathway

Following initiation of deadenylation, a series of consecutive steps results in the degradation of the target mRNA by Xrn1. As discussed above, specific steps ensure the sequential recruitment of protein complexes. Deadenylation by Pan2-Pan3 and Ccr4-Not result in the specific recognition of oligoadenylated mRNA by the Lsm1-7-Pat1 complex. It is likely that oligoadenylated mRNA is uridylated. This can assist recognition by the Lsm1-7-Pat1 complex, which prefers the presence of U residues (Montemayor et al., 2020). However, the molecular basis for the coordination of uridylation and recognition of oligoadenylated intermediates by specific terminal uridylyl transferase enzymes is unclear. In addition to specific interactions that aid the consecutive completion of the decapping and degradation events (i.e., binding of Pat1 to the Dcp1-Dcp2 complex; interaction between Dcp1-Dcp2 and the Xrn1 nuclease), a number of additional interactions have been identified between the degradation factors involved in 5′-3′ degradation. These include, for example, interactions between Lsm1-7-Pat1 and Xrn1, where the same surface of Pat1 that binds helical leucine-rich motifs in Dcp2 recognise similar motifs present in the C-terminus of Xrn1 (Bouveret et al., 2000; Chowdhury et al., 2007; Charenton et al., 2017). Pat1 can also bind to the Ccr4-Not complex (Haas et al., 2010). Moreover, the C-terminal unstructured region of Xrn1 can also interact with components of the Ccr4-Not complex (Chang et al., 2019).

In addition to multiple interactions between the core components of the 5′-3′ degradation pathway, they can also interact with other factors that module mRNA degradation. For example, the MIF4G domain of CNOT1 binds DDX6 (Dhh1/RCK/p54). This protein is an RNA helicase involved in miRNA regulation, which also activates the decapping pathway and represses translation (Chen et al., 2014; Mathys et al., 2014). Thus, multiple transient, low-affinity interactions between components of the 5′-3′ degradation pathway may result in self-organisation of factors involved in RNA degradation. The resulting local enrichment of degradation factors in the cytoplasm of eukaryotic cells may result in the formation of cytoplasmic foci known as processing bodies (P-bodies) (Van Dijk et al., 2002; Sheth and Parker, 2003; Parker and Sheth, 2007).

## Concluding remarks

In recent years, a large body of work has established critical steps in the 5′-3′ degradation pathway, including the molecular basis of the catalytic steps required for deadenylation by Pan2-Pan3, decapping, and degradation of the RNA body by Xrn1. In addition, many interactions between the molecular machines involved are understood at the molecular level. Despite immense progress, however, there are still areas that are poorly understood. For example, the requirement for the Caf1 and Ccr4 catalytic subunits during deadenylation by Ccr4-Not, and their collaborative or unique roles are not clear. In addition, the role of uridylation is not understood in detail, and the molecular basis for selective uridylation of oligoadenylated degradation intermediates is not known. A third area for future investigations is to decipher in molecular detail how events in the 5′-3′ mRNA degradation pathway are coordinated with other RNA degradation pathways and the regulation of translational efficiency.
